# Draft Genome Sequence of *Mycobacterium chelonae* Type Strain ATCC 35752

**DOI:** 10.1128/genomeA.00536-15

**Published:** 2015-05-28

**Authors:** Nabeeh A. Hasan, Rebecca M. Davidson, Vinicius Calado Nogueira de Moura, Benjamin J. Garcia, Paul R. Reynolds, L. Elaine Epperson, Eveline Farias-Hesson, Mary Ann DeGroote, Mary Jackson, Michael Strong

**Affiliations:** a Center for Genes, Environment and Health, National Jewish Health, Denver, Colorado, USA; b Computational Bioscience Program, University of Colorado Denver, School of Medicine, Aurora, Colorado, USA; c Mycobacteria Research Laboratories, Department of Microbiology, Immunology, and Pathology, Colorado State University, Fort Collins, Colorado, USA; d Department of Pediatrics, National Jewish Health, Denver, Colorado, USA; e VA Eastern Colorado Health Care System, Denver, Colorado, USA

## Abstract

*Mycobacterium chelonae* is a rapidly growing opportunistic nontuberculous mycobacterial (NTM) species that causes infections in humans and other hosts. Here, we report the draft genome sequence of *Mycobacterium chelonae* type strain ATCC 35752, consisting of 4.89 Mbp, 63.96% G+C content, 4,489 protein-coding genes, 48 tRNAs, and 3 rRNA genes.

## GENOME ANNOUNCEMENT

*Mycobacterium chelonae* is an opportunistic environmental pathogen (1) found in diverse habitats, including water, sewage, and soil (2). *M. chelonae* can cause corneal, cutaneous, and pulmonary infections in humans and other hosts. In many health care settings, the occurrence and spread of atypical mycobacterial infections, including those caused by *M. abscessus* and *M. chelonae*, have increased in prevalence in recent years. *M. chelonae* outbreaks have been attributed to factors ranging from resistance to glutaraldehyde, a commonly used hospital disinfectant (3–6) to contaminated medical equipment (7) and contaminated reagents (8).

We have sequenced and report here the first draft genome of a representative of the *M. chelonae* species. The type strain ATCC 35752 was originally isolated from the lungs of sea turtles (*Chelona corticata*), and shares phylogenetic similarity to human pathogens of this species. The genome of the *M. chelonae* type strain ATCC 35752 was assembled using 400-bp sequence reads generated from the Life Technologies Ion Torrent PGM (Thermo, Fisher Sciences, Carlsbad, CA) and 250 × 250 bp paired-end reads from the Illumina MiSeq (Illumina, Inc., San Diego, CA), at 220× coverage. Sequence reads were quality filtered using the Fastx_Toolkit (http://hannonlab.cshl.edu/fastx_toolkit/) and assembled into scaffolded contigs using the Newbler v2.9 (454 Life Sciences, Branford, CT) software package. The contigs were ordered in relation to the *M. abscessus* subsp. *abscessus* ATCC 19977 genome as a reference (9), employing the progressive Mauve 2.3.1 whole-genome alignment algorithm (10). Genomic features were annotated using the NCBI Prokaryotic Genome Annotation Pipeline (11).

The *M. chelonae* ATCC 35752 draft genome is a pseudomolecule composed of 24 contigs, with linking sequences of 100 N’s in between each contig, resulting in a total assembly size of 4,898,027 bp and a G+C content of 63.96%. The average contig length is 204,084 bp with an *N*_50_ of 462,745 bp. A total of 4,489 coding sequences (CDSs) were predicted, including 3,274 CDSs (72.93%) with functional annotations and 1,215 CDSs (27.07%) annotated as hypothetical proteins. Our genome assembly contains 48 tRNAs, 1 noncoding RNA (ncRNA), and 1 rRNA cistron consisting of the 5S, 16S, and 23S rRNA genes. A comparative analysis between *M. chelonae* ATCC 35752 and *M. abscessus* ATCC 19977 revealed 3,803 orthologous CDSs that are shared between the two strains.

Whole-genome sequence alignments of *M. chelonae* ATCC 35752 and five representative *M. abscessus* genomes revealed an average of 620,054 single nucleotide polymorphisms (SNPs), including 628,647 SNPs compared to *M. abscessus* ATCC 19977, 629,586 SNPs compared to *M. bolletii* BD^T^, 631,431 SNPs compared to *M. bolletii* M24, 621,641 SNPs compared to *M. massiliense* GO-06, and 588,967 SNPs compared to *M. massiliense* CCUG 48898 (= JCM15300). These SNP differences (12.02 to 12.85% of the *M. chelonae* ATCC 35752 genome) underscore the genomic divergence between *M. chelonae* and its sister taxa (*M. abscessus* complex).

### Nucleotide sequence accession numbers.

The draft genome sequence of *M. chelonae* ATCC 35752 has been deposited in NCBI GenBank under the accession no. CP010946, BioProject no. PRJNA251569, and BioSample no. SAMN02837161.
